# Supplementary material for: Mercury’s anomalous magnetic field caused by a symmetry-breaking self-regulating dynamo
Source: Nat Commun. 2019 Jan 14;10:208. doi: 10.1038/s41467-018-08213-7 (PMC6331596; doi:10.1038/s41467-018-08213-7)
Supplement: Supplementary file 1 — Supplementary Information [file 41467_2018_8213_MOESM1_ESM.pdf]

Supplementary Information

Mercury’s anomalous magnetic field caused by a symmetry-breaking self-regulating dynamo

Takahashi et al.

Supplementary Note 1: Co-density case

The time-averaged structures of convection and magnetic field for the model BU1C are represented in Supplementary Fig. 11. The radial field at the planetary surface is predominantly dipolar, and its intensity is much stronger than that of the BU1. Since equatorial symmetry breaking is faint, no hemispherical structure is found. Note that slight antisymmetric components exist in the velocity field, ~ 4% in kinetic energy (Supplementary Table 1). Taking into account the fact that the kinematic model (BU1K) does not have any antisymmetric flow components, it seems that a self-regulation effect must work to create the slightly asymmetric structure even in the co-density case, which is not obvious in the magnetic field at the planetary surface. Compared to the model BU1, it is suggested that double-diffusive convection actually plays a greater role in generating the asymmetric magnetic field via self-regulating effects.

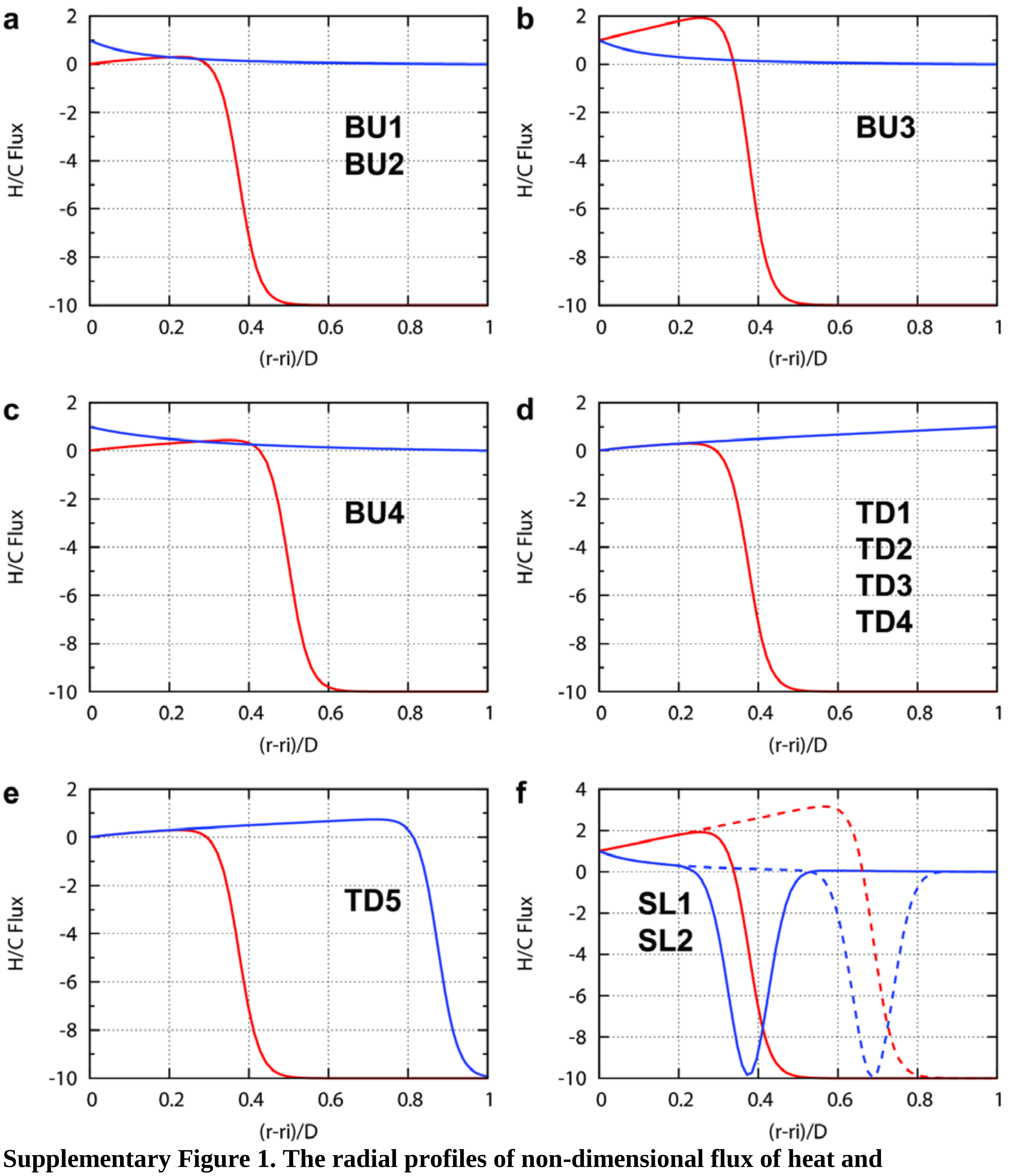

Supplementary Figure 1. The radial profiles of non-dimensional flux of heat and composition. Red lines represent the profiles of heat flux, and blue those of compositional flux. **(a)** BU1 and BU2, **(b)** BU3, (**c**) BU4 **(d)** TD1-TD4, (**e**) TD5, and **(f)** SL1 (solid lines) and SL2 (dashed lines). Positive values denote unstable stratification, while negative values denote stable stratification.

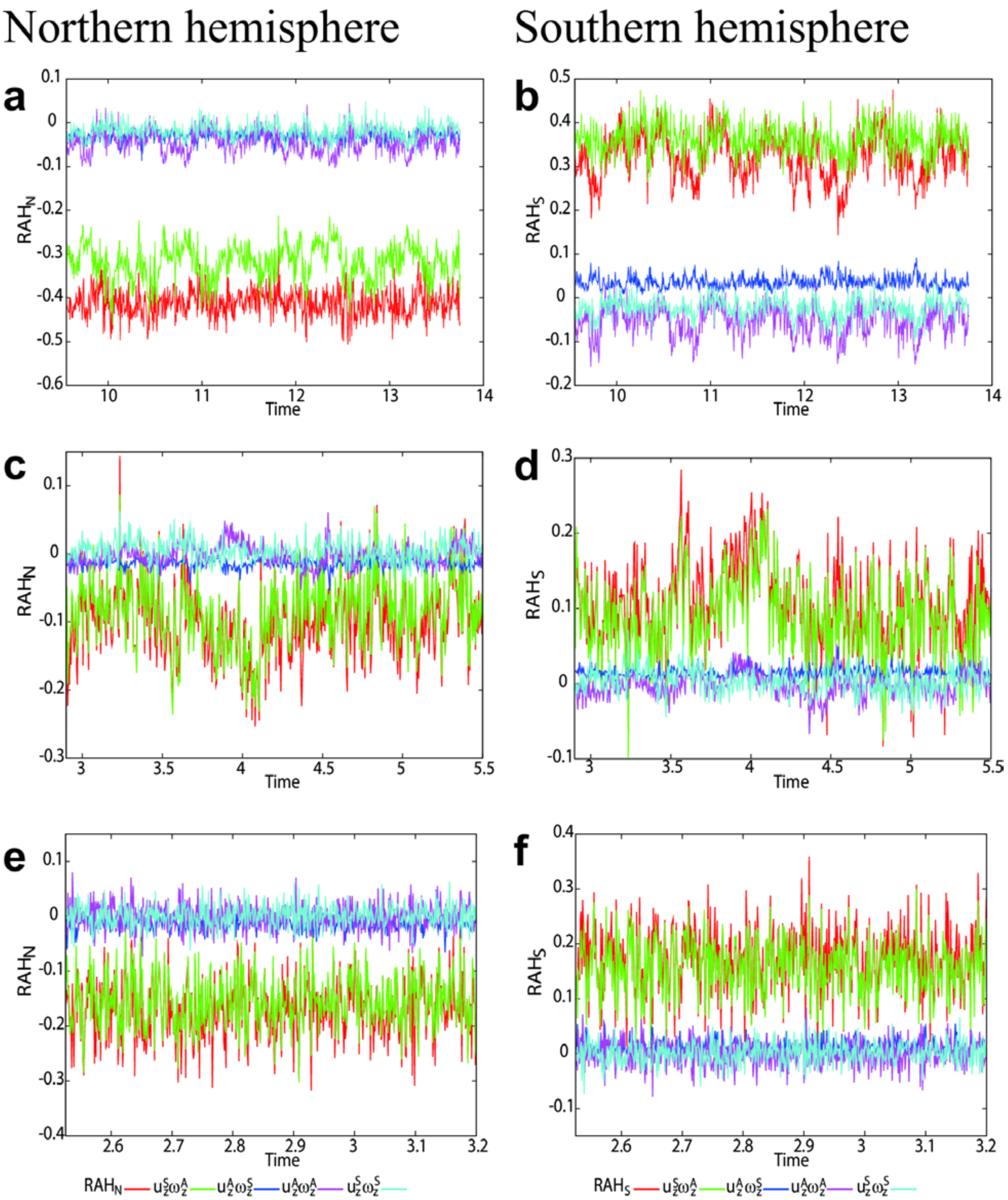

Supplementary Figure 2. Time series of the helicity partition-analysis results in each hemisphere. (**a**, **b**) for BU1, (**c**, **d**) for TD3 and (**e**, **f**) for SL1; (**a**, **c**, **e**) correspond to the northern hemisphere, and (**b**, **d**, **f**) to the southern hemisphere. Red lines represent the total values, and the others represent contributions from respective terms. Time is scaled by viscous diffusion time.

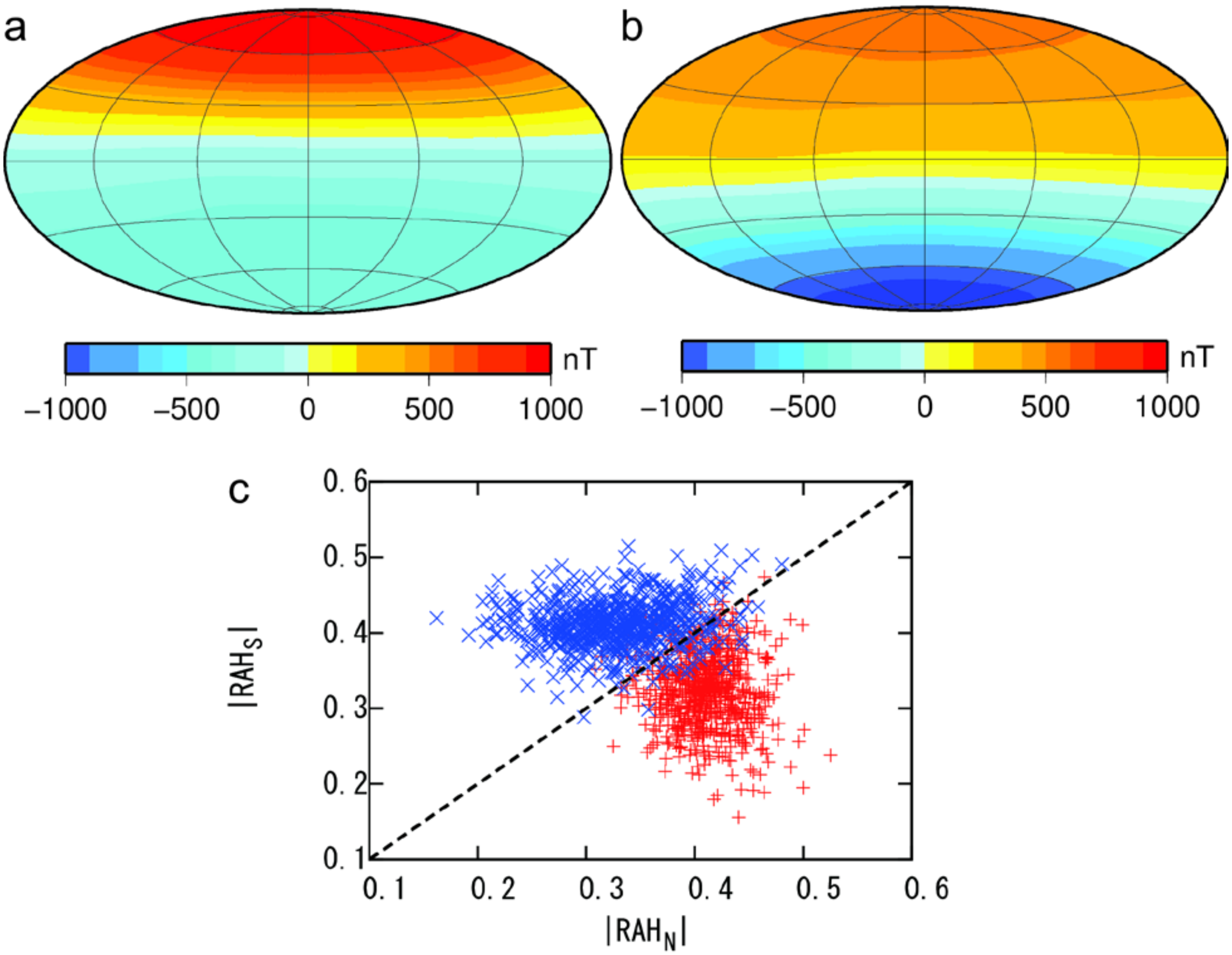

Supplementary Figure 3. Results of additional runs of BU1L and BU1M. (**a**, **b**) Snapshots of the radial magnetic field distribution at the planetary surface for (**a**) BU1L and (**b**) BU1M. (**c**) Plots of the $|RAH_N|$ vs. $|RAH_S|$. Red symbols indicate BU1L and blue BU1M.

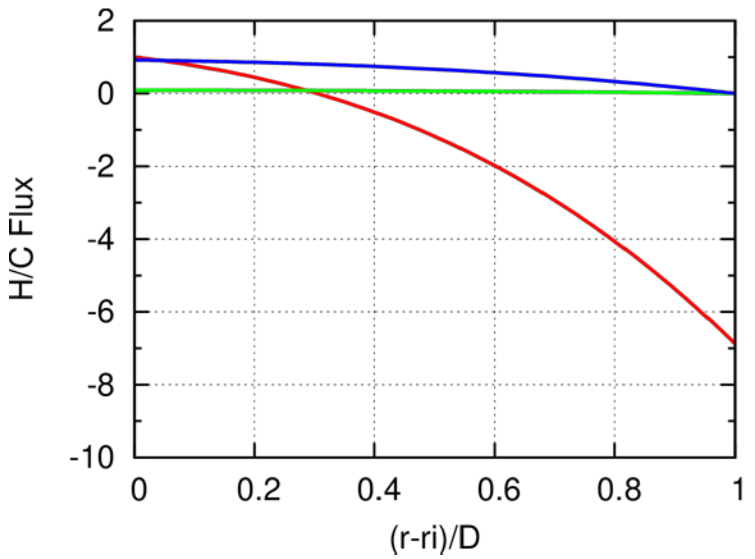

Supplementary Figure 4. Radial profiles of non-dimensional flux of heat and composition for the cases in Manglik et al. (2010). The red line represents the profile of heat flux, while relative profiles of compositional flux are shown in blue for the high-sulfur case (2%), and green for the low-sulfur case (0.2%).

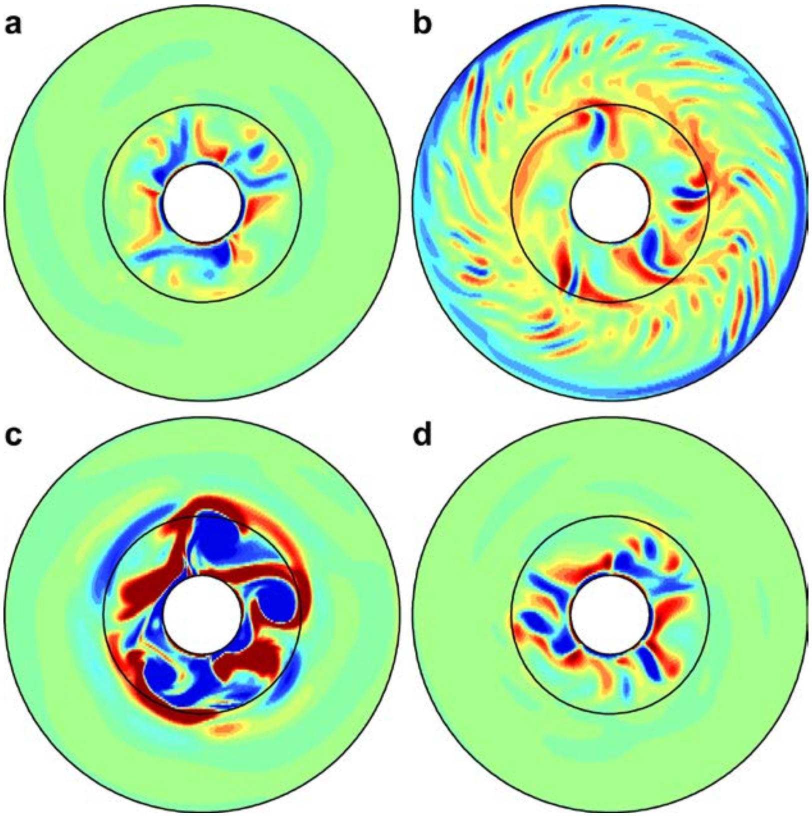

Supplementary Figure 5. Snapshots of the axial vorticity distribution on the equatorial cross-section viewed from the north. Red regions represent cyclonic vorticity, and blue regions represent anticyclonic vorticity. The outermost circle corresponds to the CMB, $r = r_o$, the middle one to $r = r_s$, and the innermost one to the ICB, $r = r_i$. **(a)** BU1, (**b)** TD1, (**c**) SL1 and (**d**) BU1K.

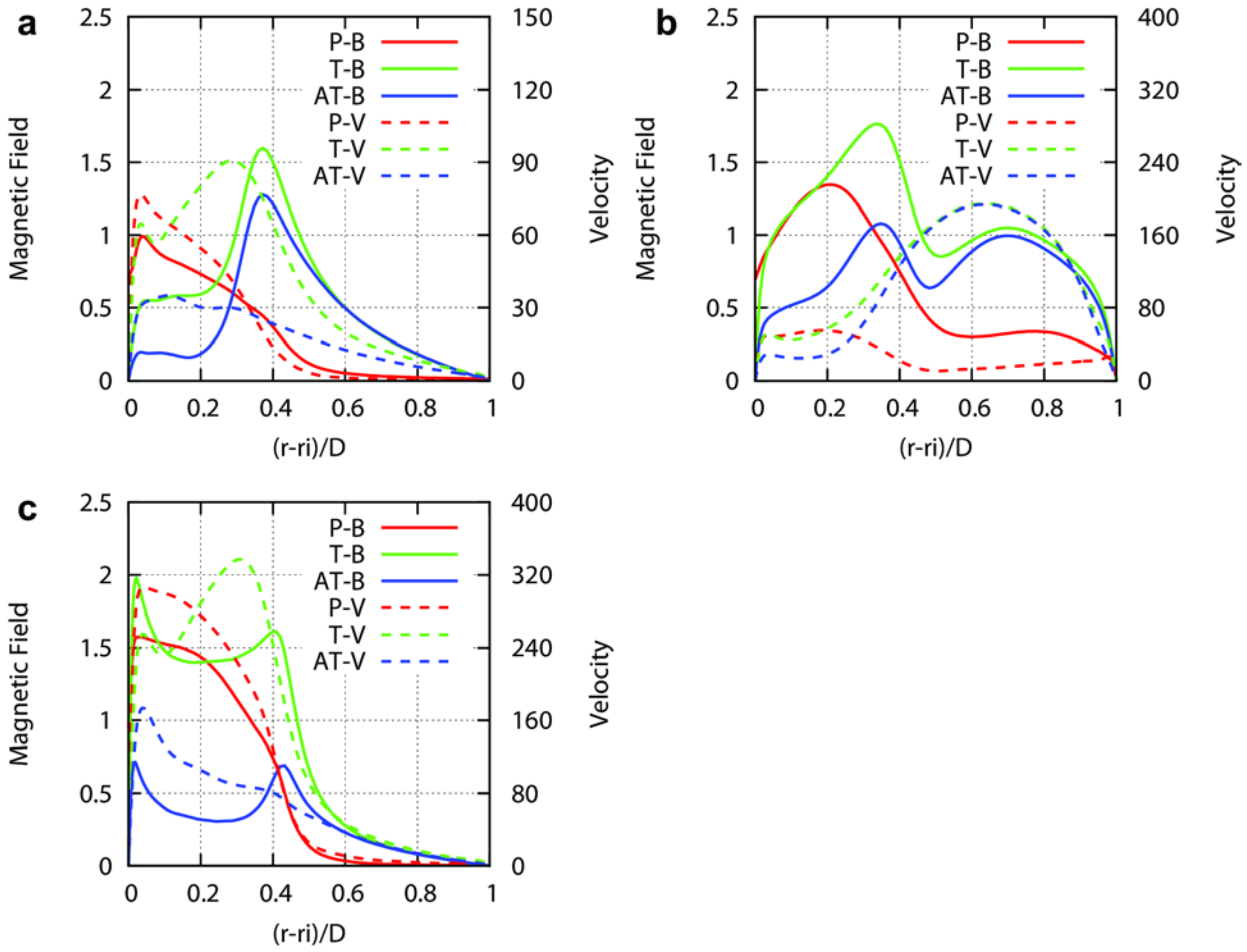

Supplementary Figure 6. Time-averaged radial profiles of root-mean-square magnetic and velocity fields averaged over spherical surfaces. Red and green lines correspond to the poloidal and toroidal components, and blue lines correspond to the axisymmetric toroidal components. The axisymmetric poloidal components are tiny and therefore not drawn. Solid (dashed) lines represent components of the magnetic (velocity) field. Stratification boundary $r = r_s$ is $r - r_i = 0.375$. **(a)** BU1, **(b)** TD1 and (**c**) SL1.

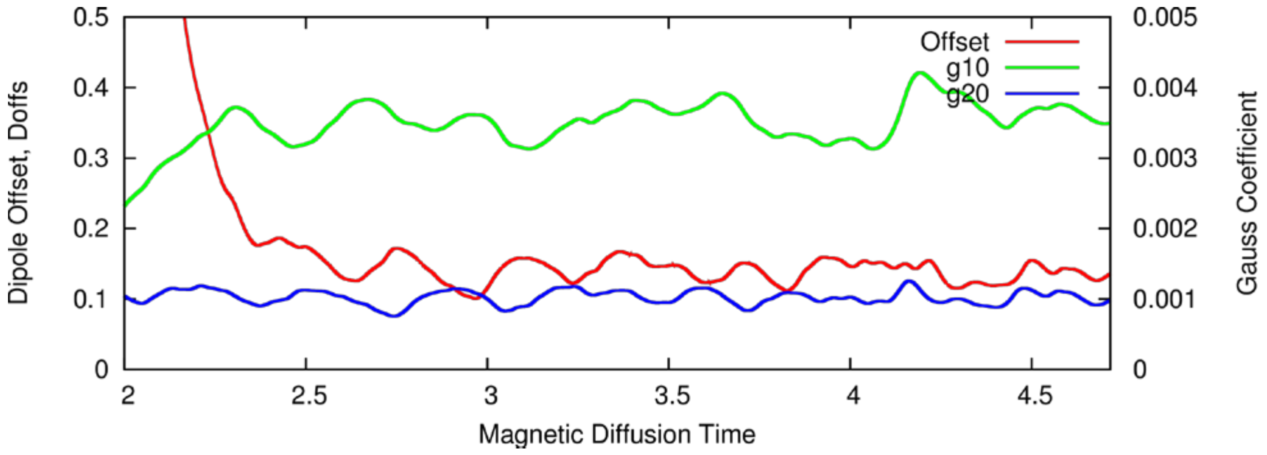

Supplementary Figure 7. Time-series of the dipole offset and Gauss coefficients of the axial dipole and axial quadrupole in BU1. The red line represents the dipole offset, D_{offs}, and the green (blue) line represents the axial dipole (quadrupole). After a transient period, a quasi-stationary state is reached.

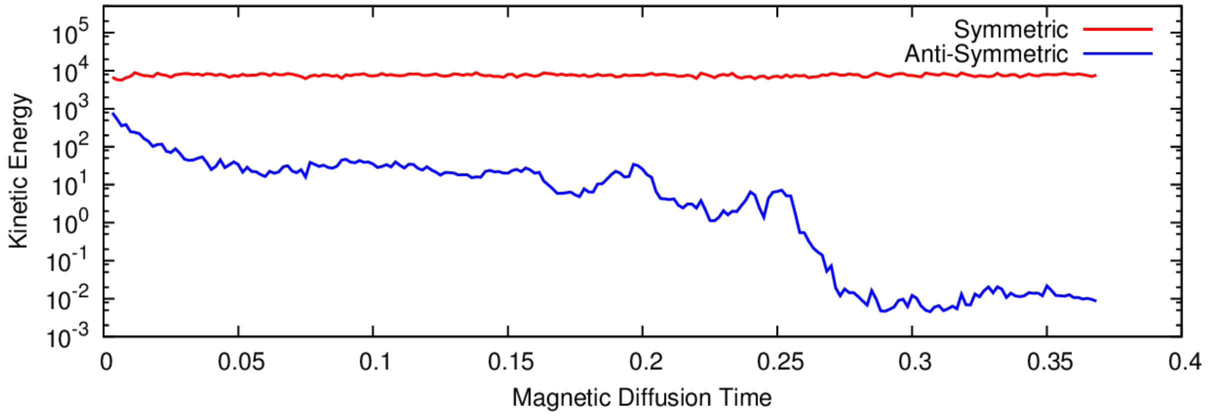

Supplementary Figure 8. Time-series of the kinetic energy density averaged over the spherical shell volume in BU1K. The red line represents the equatorially symmetric component, and the blue line represents the equatorially antisymmetric component. The antisymmetric component declines rapidly, and becomes negligible compared with its symmetric counterpart.

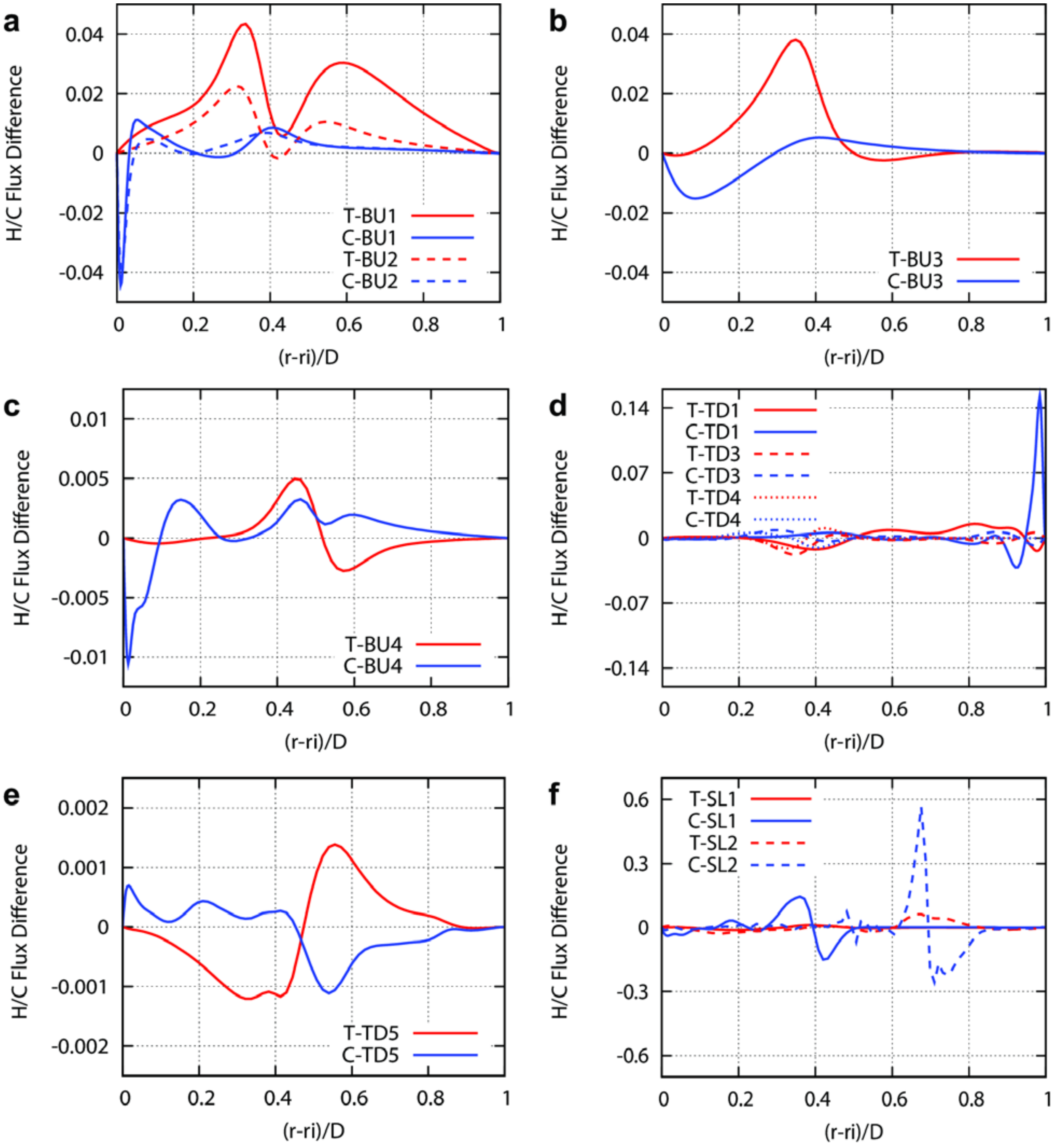

Supplementary Figure 9. Time-averaged radial profiles of non-dimensional heat and compositional flux difference between the northern and southern hemispheres. Total heat/compositional flux at the north pole minus that at the south pole is drawn. Red lines represent the profiles of heat flux difference, while those of compositional flux difference are shown in blue. **(a)** BU1 and BU2, (**b**) BU3, (**c**) BU4 (**d**) TD1, TD3 and TD4, (**e**) TD5 and (**f**) SL1 and SL2. The profiles show asymmetric distributions of the heat/compositional flux.

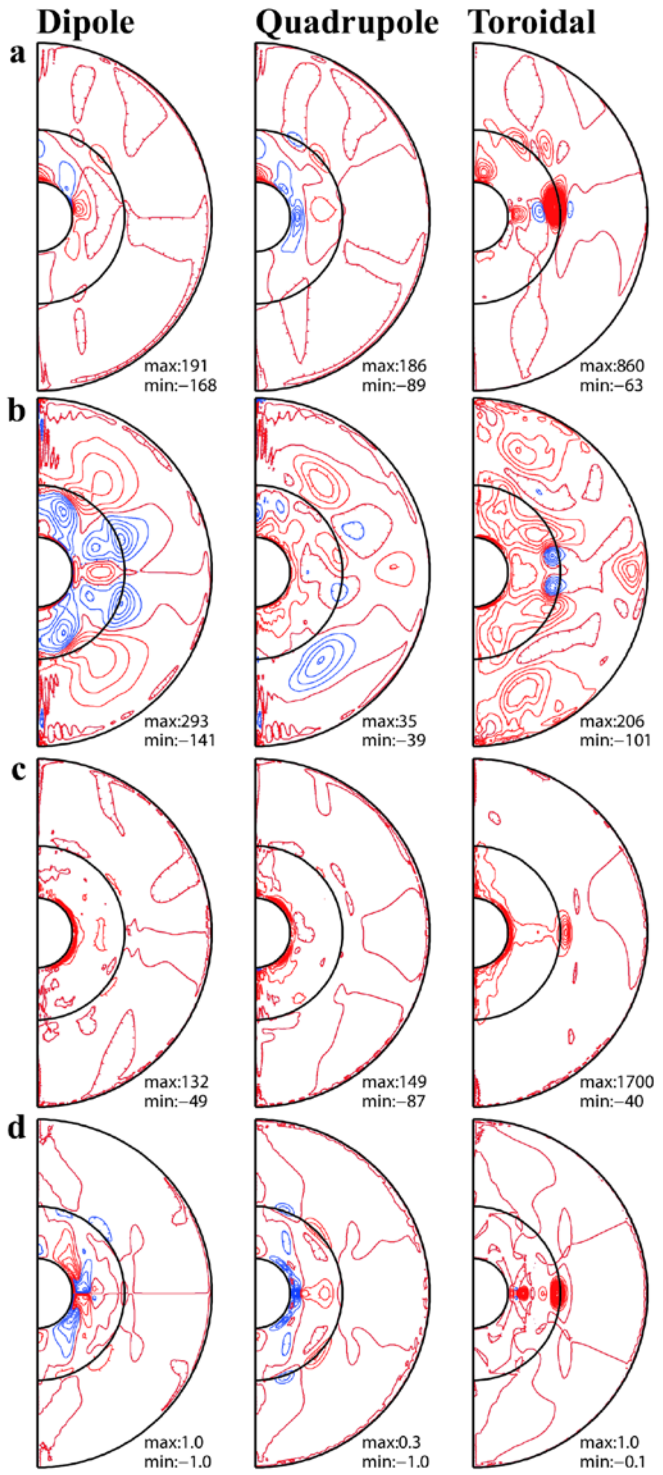

Supplementary Figure 10. Time- and azimuthally-averaged magnetic energy generation in the meridional cross section. From the left, the stretching terms for the dipole, quadrupole, and toroidal components are displayed. Red (blue) lines represents positive (negative) values. **(a)** BU1, **(b)** TD1, (**c**) SL1 and (**d**) BU1K. In (d), each component is normalized by the maximum or minimum value. The outermost circle corresponds to the CMB, $r = r_o$, the middle one to $r = r_s$, and the innermost one to the ICB, $r = r_i$.

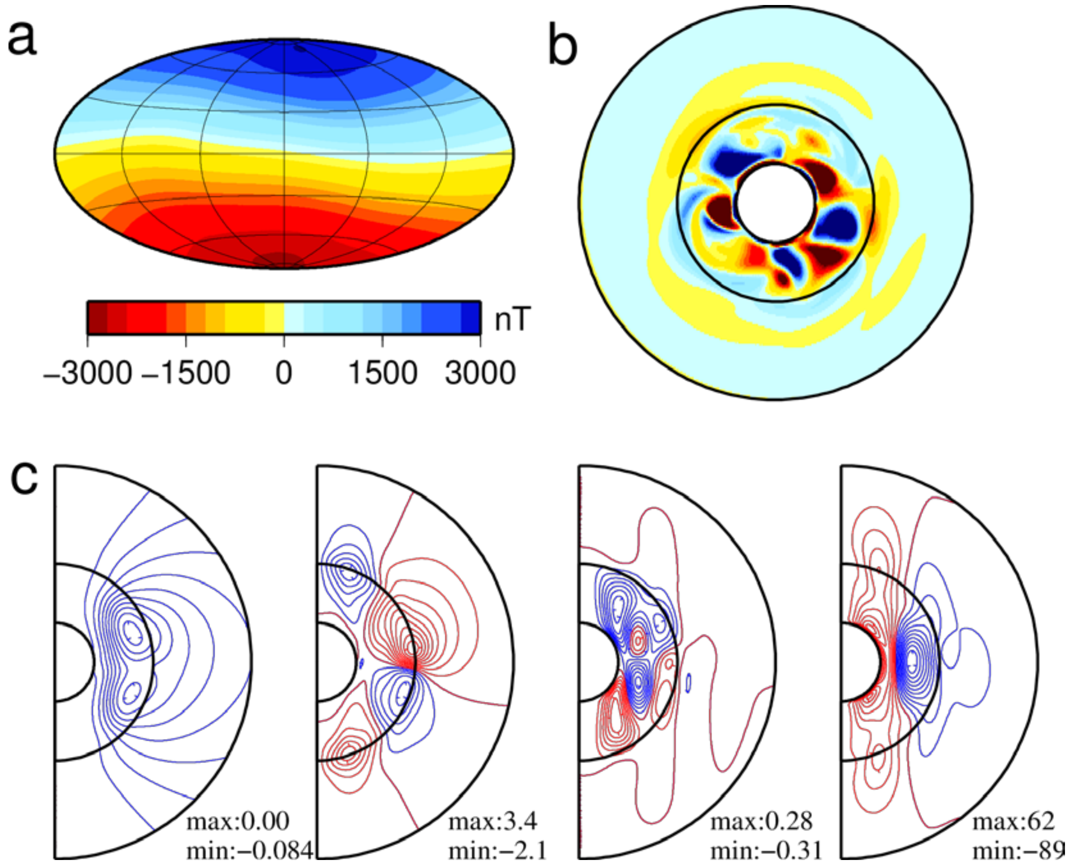

Supplementary Figure 11. Magnetic and velocity field structures in the model BU1C. (**a**) Snapshot of the radial magnetic field distribution at the planetary surface; (**b**) Snapshot of the axial vorticity distribution on the equatorial cross-section viewed from the north; (**c**) Time-averaged axisymmetric structures of non-dimensional magnetic and velocity fields in the meridional cross-section. From the left, the poloidal magnetic field, toroidal magnetic field, meridional circulation, and zonal flow are drawn, respectively, by contour lines. In (b) and (c), red (blue) regions/lines represent positive (negative) values. The outermost circle corresponds to the CMB, $r = r_o$, the middle one to $r = r_s$, and the innermost one to the ICB, $r = r_i$.

Supplementary Table 1. Time-averaged results from the dynamo simulations used in this study.

	r_s/r_o	Ra^T	Ra^C	Rm	Λ	$\lvert g_1^0 \rvert$	D_{offs}	Tilt (°)	F_{dip}	F_{axs}	K_{asym}	Ro_l	RAH_N/RAH_S
BU1	0.5	5000	10000	114	0.34	448 ± 30	0.14 ± 0.01	0.9 ± 0.6	82.0	99.9	11	0.0062	-0.42/0.33
BU1C	0.5	5000	10000	146	1.8	1180 ± 180	0.016 ± 0.004	2.8 ± 1.7	98.4	99.0	4	0.0049	-0.27/0.24
BU2	0.5	10000	10000	125	0.54	504 ± 40	0.20 ± 0.03	1.6 ± 0.7	70.7	99.9	8	0.0077	-0.38/0.29
BU3	0.5	1500	1500	112	1.05	1480 ± 100	0.02 ± 0.01	1.5 ± 0.8	98.8	99.2	2	0.0055	-0.37/0.32
BU4*	0.675	5000	5000	479	0.37	60 ± 30	0.02 ± 0.65	reversing	28.9	95.2	2	0.036	-0.23/0.22
TD1	0.5	5000	10000	436	1.27	3000 ± 240	-0.02 ± 0.01	2.4 ± 1.2	75.4	88.1	3	0.011	-0.11/0.11
TD2	0.5	10000	10000	172	na	na	na	na	na	na	0	0.010	-0.38/0.37
TD3	0.5	10000	15000	206	1.08	2800 ± 50	-0.001 ± 0.004	1.3 ± 0.6	78.8	96.9	9	0.011	-0.28/0.26
TD4	0.5	15000	15000	127	1.05	2210 ± 60	0.002 ± 0.002	0.9 ± 0.5	97.9	99.9	14	0.0066	-0.33/0.32
TD5	0.5	10000	15000	151	0.18	15 ± 13	-0.14 ± 2.44	reversing	2.3	96.5	6	0.012	-0.41./0.40
SL1	0.5	5000	250	448	0.62	32 ± 15	0.23 ± 0.16	24 ± 13	42.4	83.7	9	0.025	-0.18/0.17
SL2	0.75	1500	1500	1022	2.68	97 ± 48	-0.02 ± 0.29	reversing	54.3	78.2	11	0.054	-0.034/0.034

g_1^0 is in nT. D_{offs} is in terms of planetary radius, R_H. Tilt is a dipole tilt angle. F_{dip}, F_{axs} and K_{asym} are percentages. Ro_l is the local Rossby number. BU1C corresponds to a case of the co-density at $Pr^C = Pr^T = 0.1$. The case with an asterisk (BU4) represents a run with an Earth-like larger inner core of $\chi = 0.35$. TD2 is a case of a failed dynamo.
